# Antisense Oligonucleotide-Induced Amyloid Precursor Protein Splicing Modulation as a Therapeutic Approach for Dutch-Type Cerebral Amyloid Angiopathy

**DOI:** 10.1089/nat.2021.0005

**Published:** 2021-10-12

**Authors:** Elena Daoutsali, Tsinatkeab T. Hailu, Ronald A.M. Buijsen, Barry A. Pepers, Linda M. van der Graaf, Marcel M. Verbeek, Daniel Curtis, Thomas de Vlaam, Willeke M.C. van Roon-Mom

**Affiliations:** ^1^ Department of Human Genetics, Leiden University Medical Center, Leiden, the Netherlands.; ^2^ Amylon Therapeutics, Leiden, the Netherlands.; ^3^ Departments of Neurology and Laboratory Medicine, Radboud Alzheimer Centre, Radboud University Medical Center, Donders Institute for Brain, Cognition and Behaviour, Nijmegen, the Netherlands.; ^4^ Atalanta Therapeutics, Boston, Massachusetts, USA.

**Keywords:** amyloid precursor protein, amyloid beta, antisense oligonucleotides, Dutch-type cerebral amyloid angiopathy

## Abstract

Dutch-type cerebral amyloid angiopathy (D-CAA) is a monogenic form of cerebral amyloid angiopathy and is inherited in an autosomal dominant manner. The disease is caused by a point mutation in exon 17 of the amyloid precursor protein (*APP*) gene that leads to an amino acid substitution at codon 693. The mutation is located within the amyloid beta (Aβ) domain of APP, and leads to accumulation of toxic Aβ peptide in and around the cerebral vasculature. We have designed an antisense oligonucleotide (AON) approach that results in skipping of exon 17, generating a shorter APP isoform that lacks part of the Aβ domain and the D-CAA mutation. We demonstrate efficient AON-induced skipping of exon 17 at RNA level and the occurrence of a shorter APP protein isoform in three different cell types. This resulted in a reduction of Aβ40 in neuronally differentiated, patient-derived induced pluripotent stem cells. AON-treated wild-type mice showed successful exon skipping on RNA and protein levels throughout the brain. These results illustrate *APP* splice modulation as a promising therapeutic approach for D-CAA.

## Introduction

Dutch-type cerebral amyloid angiopathy (D-CAA), previously known as hereditary cerebral hemorrhage with amyloidosis-Dutch type, is a rare autosomal dominant disease, characterized by recurring strokes and dementia. It occurs mainly in families from two coastal villages in the Netherlands [1,2], as well as in families in Southwest Australia [3]. It is caused by a point mutation at codon 693 of the amyloid precursor protein (APP) gene resulting in a G to C change (NM_000484.3(APP):c.2077G>C). This leads to the replacement of glutamic acid with glutamine (NP_000475.1:p.Glu693Gln) within the amyloid beta (Aβ) domain of APP (E22Q).

In D-CAA, Aβ accumulates in the cerebral leptomeningeal arteries and cortical arterioles causing degeneration of vascular smooth muscle cells and ultimately leading to intracerebral hemorrhage between the ages of 40 and 65. The first hemorrhagic stroke is fatal within 1 year in one-third of the patient population, whereas surviving patients suffer from recurrent strokes and vascular dementia [4].

APP is an integral type I transmembrane glycoprotein with a large extracellular domain, a transmembrane domain, and a short cytoplasmic domain. The human *APP* gene (HGNC:620) contains 18 exons, but alternative splicing of exons 7 and 8 (encoding the Kunitz protease inhibitor and the OX-2 domains, respectively) results in three major APP protein isoforms in the brain. APP770 is the canonical isoform including all exons, while APP751 lacks exon 8 and APP695 lacks exons 7 and 8. APP695 is the predominantly expressed isoform in the brain, whereas APP770 and APP751 are found in peripheral tissues [5,6].

**FIG. 1.**
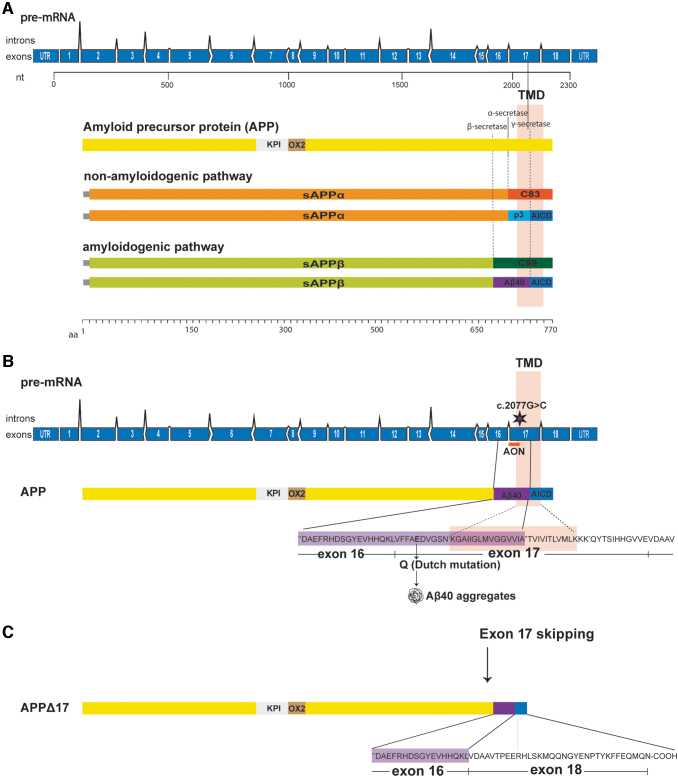
Schematic representation of APP exon 17 skipping approach. **(A)** APP proteolytic cleavage. In the nonamyloidogenic pathway, APP is cleaved by α- and γ-secretases producing sAPPα, p3, and AICD. In the amyloidogenic pathway, APP is cleaved by β- and γ-secretases generating sAPPβ, Aβ, and AICD. Black lines indicate the location of secretase cleavage sites in APP, and dashed lines the APP cleavage products. The γ-secretase cleavage site is located on exon 17. **(B)** The Dutch mutation is located in exon 17 (asterix). Also the APP transmembrane domain is coded in part by exon 17. Aβ40 is partially coded by exon 16 and exon 17. **(C)** Exon 17 skipping using an AON results in an in-frame APP transcript that is translated into the APPΔ17 protein that lacks part of the Aβ40 sequence and the transmembrane domain. Aβ, amyloid beta; AICD, APP-intracellular domain; AON, antisense oligonucleotide; APP, amyloid precursor protein; sAPPα, soluble APPα; sAPPβ, soluble APPβ; KPI, Kunitz protease inhibitor; nt, nucleotide; TMD, transmembrane domain. Color images are available online.

After mRNA translation, APP undergoes complex proteolytic processing by secretases through two major pathways generating several cleavage fragments (Fig. 1A). Based on the absence or presence of the Aβ peptide after secretase cleavage, the pathways are called nonamyloidogenic and amyloidogenic, respectively. The nonamyloidogenic pathway results in a secreted soluble APP fragment (sAPPα), the p3 fragment, and the APP-intracellular domain (AICD). In the amyloidogenic pathway, cleavage by β-secretase (BACE1) and γ-secretase results in the production of sAPPβ, the toxic Aβ peptide, and the AICD [7,8]. In CAA, the Αβ peptide that accumulates in the brain is predominantly composed of Aβ40 [9,10]. In D-CAA, it is thought that the replacement of the negatively charged glutamic acid with the neutrally charged glutamine amino acid results in accelerated oligomerization and fibrillization of Aβ as well as an impaired Aβ transport and clearance across the cerebral vasculature leading to its accumulation [11]. The Dutch mutation is not normally associated with senile plaques. Neuritic plaques and neurofibrillary tangle pathology range from very rare to absent [9,12–14].

The physiological functions of APP and its proteolytic processing products are very diverse ranging from memory and synapse function to transcriptional regulation [15].

One of the first approaches to lower APP and Αβ involved antisense oligonucleotides (AONs) injected in different transgenic Alzheimer's disease mouse models that resulted in modest behavioral improvement by decreasing APP protein levels [16,17]. That APP is important for normal brain function was shown in studies in APP knockout mice where intracerebroventricular (ICV) injection of sAPPα can increase brain repair after traumatic brain injury [18,19]. Moreover, APP knockout mice display a wide range of dysfunctions such as cerebral ischemia [20], impaired dendritic development of newborn granule cells and morphology of adult-born neurons, impaired neurogenesis after APP deletion in GABAergic neurons [21,22], as well as reduced cognitive function, behavioral abnormalities, and impaired synaptic morphology [23]. Thus, a therapeutic approach maintaining APP expression levels but removing the part of the protein that contains the D-CAA mutation would be preferable over approaches reducing APP expression.

Currently, there is only symptomatic treatment for D-CAA patients. The mutation causing D-CAA and the nucleotides that code for the γ-secretase cleavage site are both located in exon 17 of *APP* (Fig. 1B). By modulating splicing of the APP pre-mRNA using AONs, exon 17 can be removed from the APP pre-mRNA. This will result in the production of an in-frame mRNA transcript encoding for a new APP isoform that lacks 25 amino acids of the C-terminal part of the Aβ peptide including the Dutch mutation and part of the transmembrane domain (Fig. 1C).

This modified APP should maintain many of its wild-type functions but that needs to be further studied. In this study, AON treatment resulted in a shorter APP isoform (APPΔ17) based on effective modulation of the APP mRNA splicing pattern and exclusion of exon 17. In parallel with induction of APPΔ17, AON treatment resulted in the reduction of Aβ40 and Aβ42 levels in several cell models, including patient-derived induced pluripotent stem cells (iPSCs) that were differentiated into neuronal cells. Furthermore, we show modulation of APP splicing by exclusion of the equivalent exon in mice and production of the shorter APP isoform after ICV AON administration in wild-type mice. These results support the concept of splice modulation of APP as a potential therapeutic approach for D-CAA.

## Materials and Methods

### Antisense oligonucleotides

AONs were designed according to previously published protocols and guidelines [24]. The AONs used in this study were 20- and 33-mer in length with 2-methoxy ethyl (2′-MOE) and phosphorothioate (PS) backbone modifications. The AONs bind in the intron 16 and exon 17 junction of APP. The sequences of the AONs can be found in Table 1. The 20- and 33-mer AON sequences were scrambled using the online tool: Sequence Manipulation Suite; Shuffle DNA (https://www.bioinformatics.org/sms2/shuffle_dna.html); and were named 33SCR and 20SCR. All AONS were purchased from Axolabs GmbH (Germany).

**Table 1.** Antisense Oligonucleotide Sequences

Name	Sequence
APP pre-mRNA target sequence^a^	CAAGGTGTTCTTTGCAGAAGATGTGGGTTCAAAC
33AON^a^	GTTTGAACCCACATCTTCTGCAAAGAACACCTT
33SCR1	ACGAACTCTTAGTCGACTATATCTATCACGACC
33SCR2	GGCAATATACAATTTCTATCGTGCCAACCCTCA
20AON^a^	CTTCTGCAAAGAACACCTTG
20SCR1	GACTCAACCGATCTTGAACT
20SCR2	GCCACAAGCGCTTACTTAAT
Mouse 33AON	GTTCGAACCCACATCTTCAGCAAAGAACACCTT
Mouse 20AON	CTTCAGCAAAGAACACCTTC

^a^ The intron 16 sequence is shown in underlined capital letters. Exon 17 sequence is shown in capitals.

AON, antisense oligonucleotide; APP, amyloid precursor protein.

### Cell culture and AON transfection

D-CAA fibroblasts were grown from a skin biopsy from a symptomatic D-CAA patient at the Leiden University Medical Center (LUMC). The isolation of fibroblasts and transformation into iPSCs were approved by the LUMC Medical Ethical Committee (MEC) with informed consent from the patient (NL45478.058.13/P13.080). Fibroblasts were cultured in minimal essential medium (Gibco, Invitrogen, CA) supplemented with 15% fetal bovine serum (FBS) (Clontech, Palo Alto), 1% Glutamax (Gibco, California), and 1% penicillin/streptomycin (Gibco). Cells were reverse transfected with 12.5, 25, 50, and 100 nM of the 33-mer AON. Lipofectamine 2000 (Life Technologies, Carlsbad, CA) was complexed with AONs in OptiMEM serum-free medium (Thermofisher, Waltham, MA) for 20 min, according to manufacturer's instructions and then transferred to six-well plates. Cells were seeded at 4.5 × 10^5^ cells/well in a total volume of 1 mL. Serum-containing growth medium was added 4 h after transfection reaching a volume of 2 mL. Medium was refreshed with 2 mL serum-containing growth medium the next day.

Human neuroblastoma SH-SY5Y cells were maintained in a 1:1 mixture of Dulbecco's modified Eagle's medium (DMEM) (Gibco) and Ham's F-12 medium (Gibco) supplemented with 10% FBS, 1% Glutamax, and 1% Penicillin/streptomycin. SHSY5Y cells were plated at 4.5 × 10^5^ cells/well of a six-well culture plate, and were transfected with 12.5, 25, 50, and 100 nM of the 33-mer AON. AONs were complexed with Lipofectamine 2000 in OptiMEM serum-free medium (Thermofisher) for 20 min and added dropwise to the wells. OptiMEM medium was refreshed with serum-containing medium 4 h after transfection.

Human neuroblastoma SK-N-SH cells were cultured in DMEM supplemented with 10% FBS (Gibco), 0.4 mM GlutaMAX, and 1 mM sodium pyruvate (Thermofisher). SK-N-SH cells were reverse transfected with AONs using Turbofect (Thermofisher) as transfection reagent. AONs were complexed with Turbofect in OptiMEM serum-free medium (Thermofisher) for 20 min and transferred to six-well culture plates. Cells were seeded at 4 × 10^5^ cells/well in a total volume of 2 mL, and were transfected with 12.5, 25, 50, and 100 nM of the 20-mer AON. OptiMEM medium was refreshed with serum-containing growth medium 4 h after transfection.

iPSCs were cultured in mTESR1 medium (Stemcell Technologies, Vancouver, Canada) supplemented with 1% pen/strep. The generation of the iPSC lines has been previously described by our group [25]. iPSCs were differentiated to neurons, according to the protocols provided by Stemcell Technologies with minor modifications. A single cell suspension of iPSCs was plated at a density of 3 × 10^6^ cells/well in an AggreWell800 plate (Stemcell Technologies) in 2 mL of neural induction medium (NIM)+SMAD inhibitor (SMADi) (Stemcell Technologies) to form embryoid bodies (EBs). NIM+SMADi was refreshed daily for 7 days. On day 7, EBs were harvested and plated onto poly-D-lysine/laminin (PDL/laminin) (Sigma Aldrich, Missouri) coated plates in NIM+SMADi for an additional 7 days, to form neural rosettes. Medium was refreshed daily with 2 mL of NIM+SMADi. On day 14, rosette selection took place, and cells were plated in NIM+SMADi medium that was changed daily for the next 7 days. On day 21, cells were harvested and plated in NIM+SMADi medium, and 1 day later the medium was changed to neuronal precursor cell (NPC) medium (Stemcell Technologies). NPC medium was changed every other day. After four passages, NPCs were plated at a density of 5 × 10^5^ cells/well in a six-well plate for differentiation in Stemdiff Neuron Differentiation medium (Stemcell Technologies) that was refreshed daily for 7 days. Next, cells were plated for neuronal maturation in Stemdiff Neuron Maturation medium (Stemcell Technologies) that was refreshed every other day for 12 days. For the AON dose–response experiments, 5 × 10^5^ cells/well were plated in 6-well culture plates, and for the enzyme-linked immunosorbent assay (ELISA) experiment 2 × 10^5^ cells/well were plated in 12-well culture plates. Neuronally differentiated iPSCs were transfected with 12.5, 25, 50, and 100 nM of the 33-mer AON using Lipofectamine 3000 (Life technologies) as transfection reagent. AONs were complexed with lipofectamine 3000 and P3000 reagent in OptiMEM serum-free medium for 15 min and added to the wells. OptiMEM medium was refreshed with serum-containing medium 4 h after transfection.

All cell lines were maintained at 37°C and 5% CO_2_.

### Animal studies

Six to 8 weeks old male C57BL/6J mice were obtained from Charles River (Netherlands), housed and treated at Synaptologics (Netherlands). Animals were treated and cared for in accordance with Dutch regulations on the Care and Use of Laboratory Animals and approved by the VU/VUmc animal welfare commission (IvD). Mice were implanted with a unilateral guide cannula into the right lateral ventricle for repeated ICV delivery of AONs. Animals were housed individually after surgery in ventilated cages with a 12 h light/dark cycle. Food and water were available *ad libitum*. For AON injections, a total of four mice per group, aged 8–10 weeks, were used. AONs were prepared in Dulbecco's phosphate-buffered saline (DPBS) and sterile filtered in a 0.20 μM filter. AON injections were performed under isoflurane anesthesia (2%) at a flow rate of 0.5 μL/min for total volume of 10 μL over 20 min. The desired final AON dose was achieved through repeated infusions under isoflurane anesthesia separated by 2 to 3 days. Mice were sacrificed 7 days after the last injection. Brain tissue was collected, dissected by region, snap frozen in liquid nitrogen, and stored at −80°C until further use. Cortex, hippocampus, midbrain, striatum, cerebellum, olfactory bulb, spinal cord, and kidney were dissected from the animals. For RNA isolation, tissue was first disrupted and homogenized in lysis buffer using MagNA Lyser Green Beads in a MagNA Lyser Instrument (Roche, Basel, Switzerland). Total RNA was isolated using RNeasy mini kit (Qiagen, Hilden, Germany) according to the manufacturer's instructions. RNA was eluted using nuclease-free water from columns and stored at −80°C after adjusting concentrations to 100 ng/μL. For protein isolation, the N-PER Neuronal Protein Extraction Reagent (Thermofisher) was used according to manufacturer's instructions.

### RNA analysis

For the RNA analysis in the *in vitro* dose–response experiments, RNA was isolated 24 h after transfection by trypsinizing the cells and collecting the cell pellet. For the confirmation of exon 17 skipping in neuronally differentiated iPSCs in the Aβ40 ELISA experiments, RNA was isolated 4 days after transfection.

Fibroblasts, SHSY5Y cells, SK-N-SH cells, and neuronally differentiated iPSCs were lysed, and the Reliaprep RNA Cell Miniprep kit (Promega, Madison) was used for RNA extraction, according to manufacturer's instructions. RNA was eluted using nuclease-free water and stored at −80°C. For fibroblasts, SHSY5Y cells, and neuronally differentiated iPSCs, 500 ng RNA was used as input for cDNA synthesis using the Transcriptor First Strand cDNA Synthesis kit (Roche, Mannheim, Germany), according to manufacturer's instructions. Exon skipping was assessed using the Expand High Fidelity PCR kit (Roche, Basel, Switzerland). Two microliters of cDNA, 200 μM of each deoxynucleotide, 500 nM of forward (exon 16) and reverse (exon 18) primer, 1.7 U expand high-fidelity polymerase and polymerase chain reaction (PCR)-grade water were added to a final volume of 25 μL. The PCR program consisted of an initial denaturation step at 95°C for 4 min, followed by 40 cycles of denaturation at 95°C for 10 s, annealing at 60°C for 30 s, and elongation at 72°C for 20 s and a final elongation step at 72°C for 7 min. All primers are listed in Table 2. Reaction products were loaded into a 1.5% agarose gel with ethidium bromide, and pictures were captured with the Optigo system (Isogen Life Science, Netherlands).

**Table 2.** Polymerase Chain Reaction Primer Sequences

Target gene	Primer name	Sequence (5′ to 3′)
hAPP	hAPPex14F	CGTCTTGGCCAACATGATTA
hAPP	hAPPex18R	TTGGATTTTCGTAGCCGTTC
hAPP	AT01-Pr_009	TCCGACATGACTCAGGATATGA
hAPP	AT01_Pr_010	TAGCCGTTCTGCTGCATCTT
mAPP	AT01-Pr_028	TCTGGGCTGACAAACATCAA
mAPP	AT01_Pr_029	TTCTGCTGCATCTTGGAGAG

hAPP, human amyloid precursor protein; mAPP, mouse amyloid precursor protein.

For SK-N-SH cells and mouse brain samples, cDNA was synthesized with Maxima or Verso cDNA synthesis kits (Thermofisher) according to the manufacturer's instruction using anchored Oligo dT or a gene-specific primer. PCR was performed using AmpliTaq Gold 360 DNA Polymerase kit (Thermofisher) according to the manufacturer's instructions. PCR fragments were analyzed on the 2100 Bioanalyzer Instrument (Agilent, CA).

The exon-skipped bands were quantified using ImageJ software for the RNA blots and the Image Studio Lite Software for the western blots (LI-COR, Lincoln, NE). For the *in vivo* study, the skipped band was quantified using the 2100 Bioanalyzer Instrument Software (Agilent, CA). Exon 17 skipping efficiency was calculated as a fraction of the skipped band to the total APP (APPΔ17/(APPΔ17+fl-APP)*100).

### Protein analysis

Fibroblasts, SH-SY5Y cells, and neuronally differentiated iPSCs were trypsinized 48 h after transfection; cell pellets were washed twice with phosphate-buffered saline (PBS) (Gibco) and resuspended in RIPA lysis buffer (50 mM Tris-HCl pH 8.0, 150 mM NaCl, 1% IGEPAL CA-630, 0.5% DOC, 0.1% SDS) supplemented with cOmplete EDTA-free protease inhibitors (Roche, Basel, Switzerland). Samples were incubated for 30 min in a head-over-head rotor at 4°C, centrifuged for 10 min at 10,000 rpm, and fast frozen with ethanol at −80°C. For neuronally differentiated iPSCs, an additional step of sonication was included in the protocol after lysis. Neuronally differentiated iPSCs were disrupted by sonicating 10 times at an amplitude of 60 Hz for 30 s at 4°C.

For SK-N-SH cells, protein was isolated using M-PER Mammalian Protein Extraction Reagent (Thermofisher). Protein concentrations for all cell lines were determined using the BCA kit (Thermofisher) according to manufacturer's instructions.

Protein samples were separated using a 12% precast sodium dodecyl sulfate polyacrylamide gel (Bio-Rad, Hercules, CA) for mouse brain lysates or a 4%–20% electrophoresis gel for cell culture lysates. The Transblot Turbo System (Bio-Rad) was used for protein blotting onto a 22 μm filter Trans-Blot Turbo nitrocellulose membranes (Bio-Rad).

For details on antibodies used, see Table 3. For the fibroblasts, SH-SY5Y and neuronally differentiated iPSCs membranes were blocked with 4% low-fat milk in tris-buffered saline (TBS) for 45 min. Then, membranes were probed with the primary antibodies: Y188 that is specific for the C-terminal region of APP, at a 1:5,000 dilution and beta-actin that was used as a loading control at a 1:10,000 dilution. After three washing steps with TBS, the membranes were incubated with secondary antibodies for 1 h; goat antimouse IRDye 680RD or goat antirabbit IRDye 800CW at a 1:5,000 dilution. Bands were visualized using the Odyssey Infrared imaging system (LI-COR).

**Table 3.** Antibodies

Antibody name	Dilution	
Aβ precursor protein antibody [Y188]	1:2,000	Abcam Cat# ab32136, RRID:AB_2289606
Beta-actin	1:10,000	Abcam Cat# ab6276, RRID:AB_2223210
Goat antirabbit IgG, IRDye 680 conjugated antibody	1:5,000	LI-COR Biosciences Cat# 926-32221, RRID:AB_621841
Goat antimouse IgG, IRDye 800CW conjugated antibody	1:5,000	LI-COR Biosciences Cat# 926-32221, RRID: AB_2782997

Aβ, amyloid beta.

For SK-N-SH cells and mouse brain tissue samples, membranes were washed in double distilled water and blocked in Odyssey Blocking Buffer (LI-COR) for 1 h at room temperature. Membranes were then washed once in PBS and incubated overnight in blocking buffer containing Y188 diluted 1:4,000 at 4°C. Membranes were then washed three times for 5 min in 0.2% PBS-Tween 20 and 1 time for 5 min in PBS, and incubated for 1 h at room temperature with appropriately diluted IR-Dye secondary antibody. Following this, membranes were washed three times for 10 min in 0.2% PBS-Tween 20 and one time in PBS, dried sandwiched in filter paper, and imaged on an Odyssey CLx near-infrared imaging system (LI-COR). The APPΔ17 isoform was quantified using the Image Studio Lite Software (LI-COR).

### Enzyme-linked immunosorbent assay

For Aβ40 ELISA in neuronally differentiated iPSCs and SK-N-SH cells, the Human/Rat β-Amyloid (40), Wako kit (Fujifilm), and Aβ 40 Human ELISA Kit (Thermofisher) were used, respectively, following manufacturer's instructions. For Aβ40 ELISA from conditioned media of SK-N-SH cells, samples were concentrated using 3 KDa cutoff Amicon Untra (Merck, Darmstadt, Germany) centrifugal units to enable detection within the dynamic range of the assay. Aβ40 and Aβ42 were quantified in cell supernatants of neuronally differentiated iPSCs using specific ELISAs on the semiautomated Lumipulse system (Fujirebio, Tokyo, Japan). sAPPα was quantified using an ELISA (IBL, Tecan, Männedorf, Switzerland) according to the manufacturer's instructions, with the only exception that 50 μL sample volume was used instead of 100 μL [26].

### Statistical analysis

Statistical analyses were performed using GraphPad Prism 8.1.1. For ELISAs comparing more than two groups, one-way analysis of variance (ANOVA) with a *post hoc* test (Tukey) was used. For ELISAs where two independent variables were compared, two-way ANOVA with a *post hoc* test (Bonferroni) was used to assess significant differences. The statistical test used for each experiment is mentioned in the figure legends.

## Results

### Thirty-three-mer AON induces exon 17 skipping in multiple cell lines *in vitro*

**FIG. 2.**
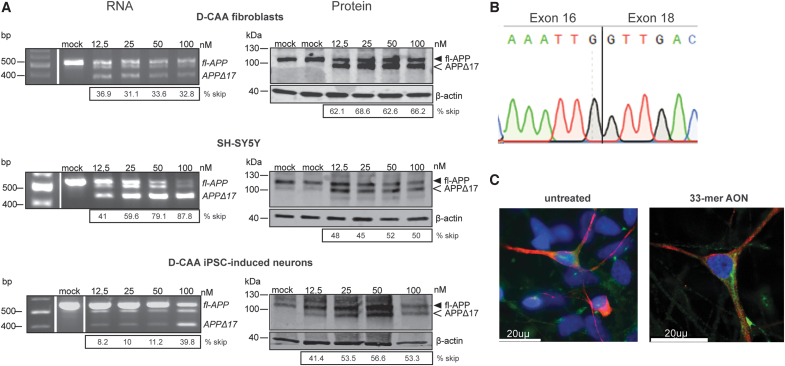
The 33-mer AON successfully modifies APP on RNA and protein levels *in vitro*, in different cell lines. *In vitro* 33-mer AON-induced exon 17 skipping **(A)** RNA and protein analysis of 33-mer with increasing concentrations of AON in patient fibroblasts, SHSY5Y and patient iPSC-induced neurons, compared with mock-transfected cells. fl-APP and APPΔ17 refer to APP mRNA and protein that include or lack exon 17, respectively; **(B)** Sanger sequencing of the exon-skipped band showing the removal of exon 17 after AON treatment. **(C)** Immunofluorescent staining with antibodies against full-length APP (Y188 antibody) and Aβ (4G8 antibody) in untreated and 33-mer AON treated neuronally induced iPSCs. fl-APP, full-length APP; iPSC, induced pluripotent stem cell. Color images are available online.

**FIG. 3.**
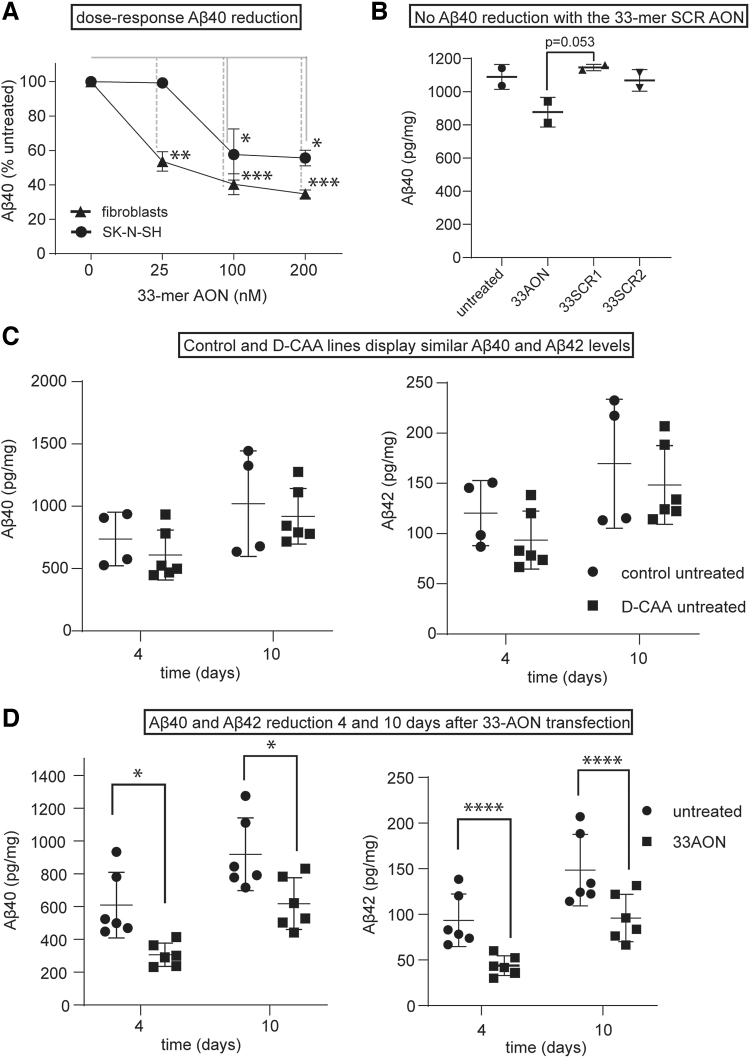
The 33-mer AON reduces Aβ levels in culture media of different cell lines. **(A)** Aβ40 ELISA analysis (Aβ 40 Human ELISA Kit) (Thermofisher, Waltham, MA) of increasing concentrations of 33-mer AON treatment of control fibroblasts and SK-N-SH cell 24 h after transfection. Aβ40 levels were measured in the culture medium and are expressed as a percentage of the Aβ40 levels in mock-transfected cells. **(B)** Aβ40 ELISA analysis (Human/Rat β-Amyloid (40) Wako kit (Fujifilm) of untreated cells, 100 nM 33-mer AON, 33-mer SCR1 and 33-mer SCR2 treatment in control neuronally differentiated iPSCs. Aβ40 was measured in the culture medium and Aβ40 levels were normalized to total protein levels, as measured by BCA. **(C)** Aβ40 and Aβ42 ELISA analysis (Lumipulse ELISA) in untreated cells from control and patient neuronally differentiated iPSCs. Aβ40 and Aβ42 levels were measured in the cultured medium and were normalized to total protein levels, as measured by BCA. **(D)** Aβ40 and Aβ42 ELISA analysis (Lumipulse ELISA) of 100 nM 33-mer AON treatment in patient neuronally differentiated iPSCs, compared with untreated cells, 4 and 10 days after transfection. Aβ40 and Αβ42 levels were measured in the culture medium and were normalized to total protein levels, as measured by BCA (±SD; *P < 0.05, **P < 0.01, ***P < 0.001, ****P < 0.0001, two-way ANOVA with Bonferroni's multiple comparisons test; *n* = 6). ANOVA, analysis of variance; BCA, bicinchoninic acid assay; ELISA, enzyme-linked immunosorbent assay; SD, standard deviation.

After AON transfection, all cell lines showed efficient exon 17 skipping on RNA and protein level (Fig. 2A). Alongside the 33-mer AON, a FAM-labeled AON was used to assess the transfection efficiency, which was in all cases between 70% and 90%. AON transfection in D-CAA fibroblasts showed a prominent skip on RNA level at all concentrations (ranging from 31.1% to 36.9%), while in both SH-SY5Y cells and D-CAA neuronally differentiated iPSCs a dose-dependent increase in exon 17 skipping was observed with a simultaneous decrease of the APP-wt transcript (41%–87.8% and 8.2%–39.8%, respectively). On protein level, D-CAA fibroblasts and SH-SY5Y cells showed a very efficient exon 17 skipping that was similar across all AON concentrations (62.1%–68.6% and 48%–52%, respectively). In D-CAA neuronally differentiated iPSCs, a dose-dependent increase of modified APPΔ17 protein was observed (41.4%–56.6%). The skipped APPΔ17 product was quantified as a fraction of the total APP for both RNA and protein. Sanger sequencing of the exon-skipped PCR product confirmed successful removal of exon 17 (Fig. 2B). In general, all cell lines showed minimum or no signs of cell death after AON transfection based on microscopy observation. Exon skipping efficiency was variable between the different cell lines most likely due to cell-line-dependent APP expression levels. Immunofluorescent staining of D-CAA neuronally differentiated iPSCs with an antibody targeting fl-APP (Y188) and an antibody targeting Aβ (4G8) did not show any overt changes in cell morphology and APP localization (Fig. 3C).

### Exon 17 skipping reduces Aβ40 levels *in vitro*, in three different cell lines

To examine if removal of exon 17 of the APP pre-mRNA reduces Aβ production, the levels of Aβ40 were measured using different ELISAs (see Methods section). First, we studied the effect of increasing dose of AON on Aβ40 levels. Transfection of control fibroblasts and SK-N-SH cells with increasing concentrations of 33-mer AON resulted in a dose-dependent decrease of Aβ40. Treating the cells with 100 nM 33-mer AON led to a 40% reduction of Aβ40. The values were normalized to mock-transfected cells (Fig. 3A). A concentration of 100 nM was chosen to continue with the follow-up ELISAs. Next, we investigated if the Aβ40 reduction is a specific effect of the 33-mer AON. A clear trend in decreased Aβ40 levels was found 4 days after 33-mer AON transfection, whereas Aβ40 levels transfected with 33-mer SCR1 and 33-mer SCR2 AONs were similar to untransfected cells (Fig. 3B). Finally, we investigated the effect of transfection of the 33-mer AON in neuronally differentiated iPSCs on Aβ40, Aβ42, and sAPPα levels at different time points. First, we measured the basal Aβ40, Αβ42, and sAPPα levels in control and D-CAA neuronally differentiated iPSCs (Fig. 3C), to confirm that there is no increased production of Aβ40 in the D-CAA. Both at 4 and 10 days after AON transfection, the levels of Aβ40 and Aβ42 were significantly lower, compared with untransfected cells (Fig. 3D). Although our exon skipping approach retains the APP alpha secretase cleavage site in APPΔ17 (Fig. 1), we measured a similar reduction in sAPPα levels, as was found for Aβ40 and Aβ42, 4 and 10 days after AON transfection compared with untreated samples (Supplementary Fig. S1). All values were normalized to total protein level. We confirmed the reduction in Αβ levels using different nonautomated and automated ELISAs, and although they detected different total levels of Aβ, the reduction of AON treatment was very consistent across assays.

### AON-mediated exon skipping in wt-mice

**FIG. 4.**
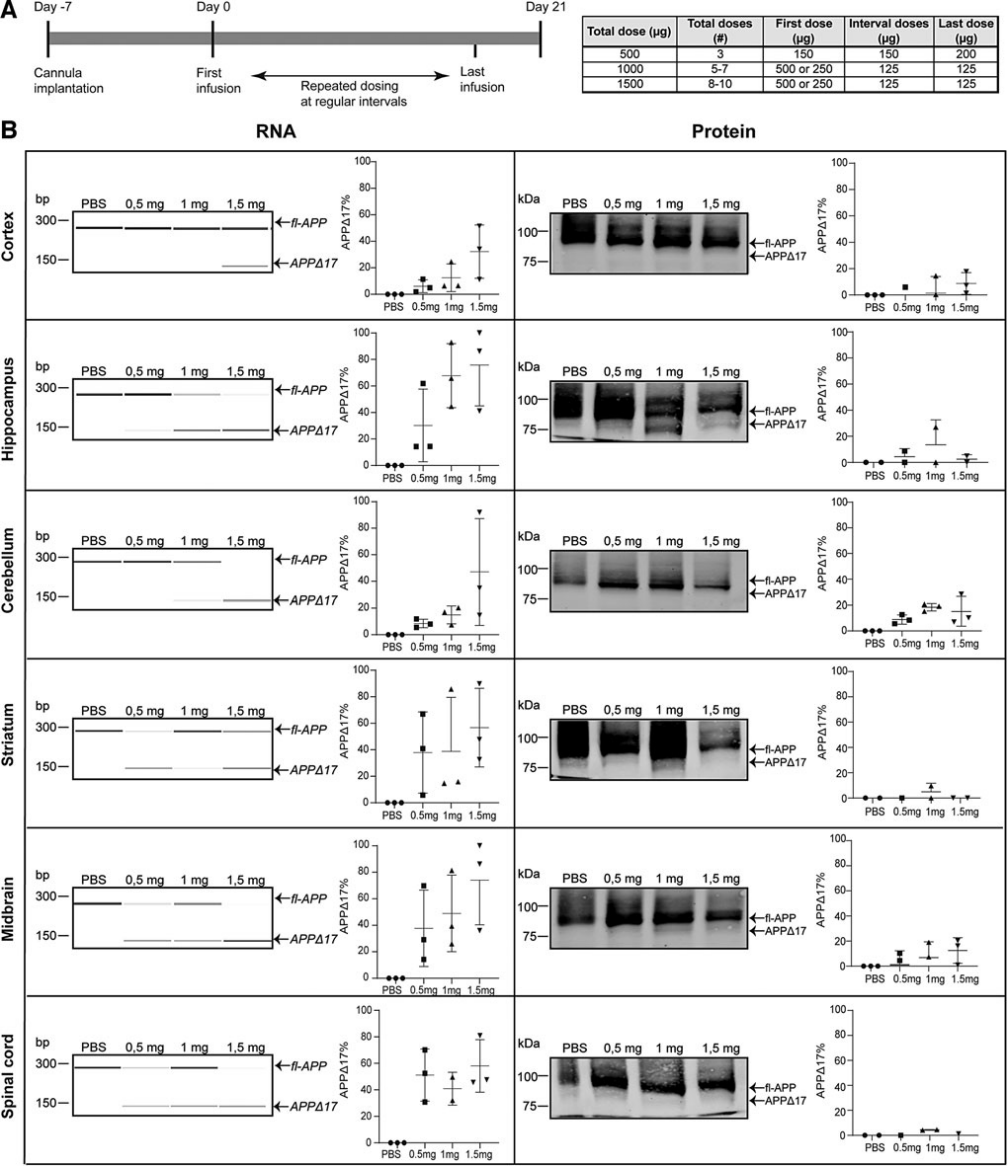
The 33-mer AON successfully modifies APP on RNA and protein level *in vivo*. **(A)** Schematic representation of the *in vivo* study timeline and the dosing scheme. **(B)** APP RNA (*left*) and protein (*right*) analysis of 33-mer AON dose–response (0.5 mg, 1 mg, and 1.5 mg) treatment in wt-mice. The different rows represent different brain areas isolated from AON-treated and PBS-treated mice. Representative PCR and western blot data from different brain regions from one mouse per treatment group are shown (PBS treated, 0.5 mg, 1 mg, and 1.5 mg AON treated). The graphs represent the exon-skipped band as a percentage of the total APP of all animal groups (three mice/treatment group) together. Error bars are median ± SD. The skipped band was quantified using the 2100 Bioanalyzer Instrument Software (Agilent). PBS, phosphate-buffered saline; PCR, polymerase chain reaction.

To test if the 33-mer AON can induce exon skipping *in vivo*, the AON was made complementary to exon 15 of the mouse APP mRNA that is homologous to the human exon 17 of APP and was delivered into the cerebral ventricles of nine wild-type mice. Three wt-mice were treated with PBS. RNA and protein were isolated 21 days after AON administration. The timeline and dosing regimen of the *in vivo* study can be found in Figure 4A. On RNA level, a dose-dependent exon skip in most of the brain regions was found (Fig. 4B), in all treated animals (Supplementary Fig. S2). There is variability in exon skipping efficiency on RNA level between the different brain areas; in midbrain, striatum, and cerebellum there is a skipping efficiency of 60%–80%, whereas in cortex it is <40%. In some conditions, the PCR product representing the unskipped APP is reduced but differs between different doses and brain areas (Supplementary Fig. S2). On protein level, exon skipping was observed only in hippocampus and striatum, with 1 mg and 1.5 mg AON concentrations, whereas in the rest of the brain areas and animal groups little or no protein modification was observed (Fig. 4B and Supplementary Fig. S2). The APPΔ17 protein was quantified as a percentage of the total APP and background was subtracted based on the PBS values.

### Shorter AON sequence leads to more efficient exon skipping in wt-mice

**FIG. 5.**
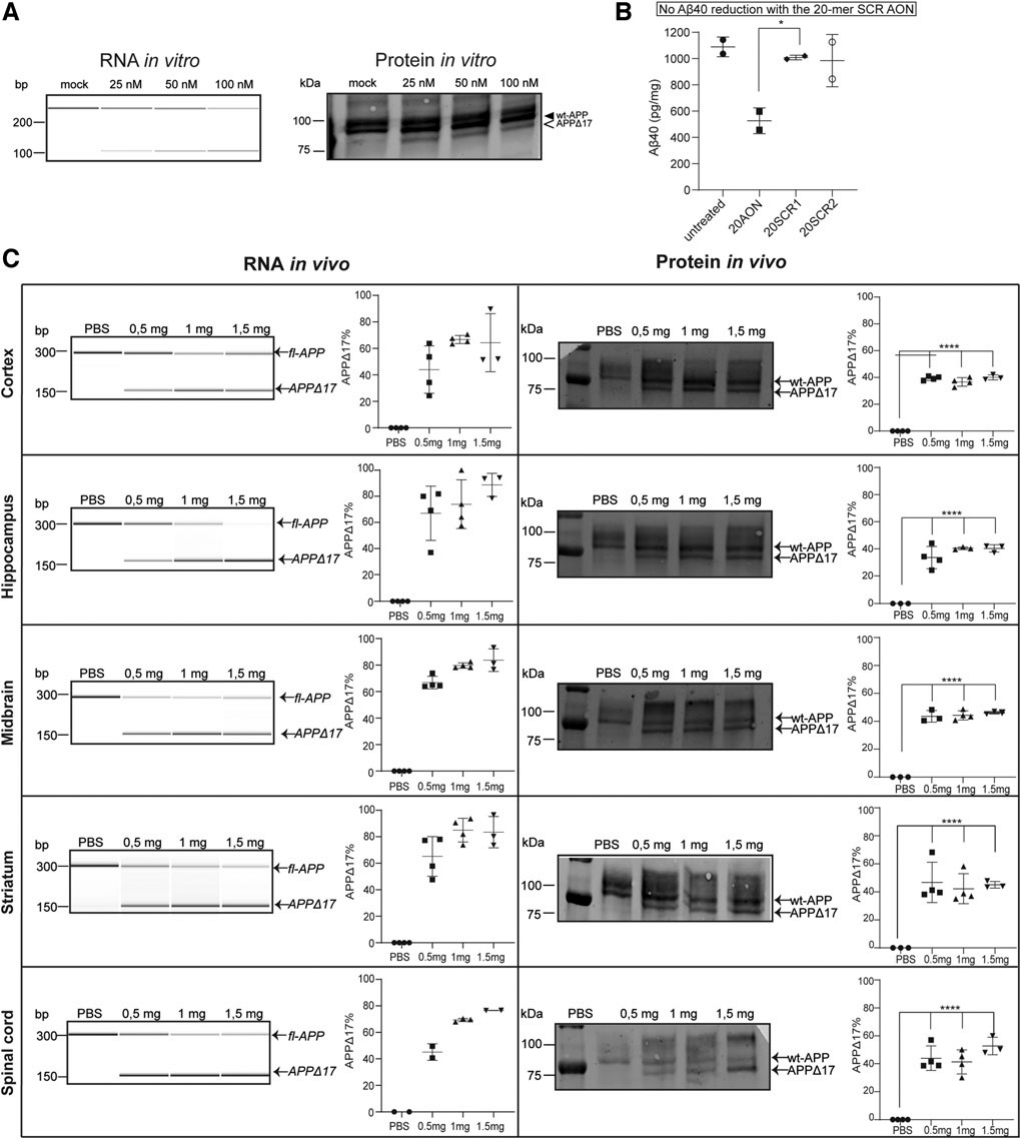
The shorter 20-mer AON leads to a greater reduction of Aβ40 *in vitro* and a more efficient APPΔ17 generation *in vivo*. **(A)** RNA and protein analysis of increasing concentrations of 20-mer AON treatment in SK-N-SH cells. Exon 17 skipping efficiency was calculated as a fraction of the skipped band to total APP. fl-APP and APPΔ17 refer to APP mRNA and protein that include or lack exon 17, respectively. **(B)** Aβ40 ELISA analysis of 100 nM 20-mer AON, 20-mer SCR1, and 20-mer SCR2 compared with mock- and 33-mer AON-transfected cells. Aβ40 levels were measured in the culture medium and were normalized to total protein levels, as measured by BCA (±SD; *P < 0.05, ****P < 0.0001, one-way ANOVA with Tukey's multiple comparisons test; *n* = 2). **(C)** *In vivo* RNA (*left*) and protein (*right*) analysis of 20-mer AON dose response from one mouse per treatment group is shown (0.5 mg, 1 mg, and 1.5 mg) treatment in wt-mice. The different rows represent different brain areas isolated from the treated and PBS-treated mice. The graphs next to the PCR gel show quantitation of the exon 15 skipping of all animal groups (four mice/treatment group). Error bars are median ± SD.

Since the effect observed with the 33-mer AON *in vivo* was promising but the protein modification modest, we designed a shorter 20-mer AON. First, we tested the efficiency of the 20-mer AON in cultured cells. *In vitro* exon 17 skipping in SK-N-SH cells with the 20-mer AON was observed on both RNA and protein levels, in a dose-dependent manner (Fig. 5A). Next, we assessed the ability of the 20-mer AON to reduce Aβ40 levels. The 20-mer AON led to a 50%–60% Aβ40 reduction compared with untreated and to a 20% reduction in the 33-mer AON-treated cells. Moreover, two different 20-mer SCR AONs were taken along in the experiment to ensure reduction in Aβ40 levels was due to a specific effect of the AON. There was no significant reduction of Aβ40 in the 20-mer SCR AON-treated cells. All values were normalized to total protein levels (Fig. 5B). There was no increased cell death observed after AON and SCR AON transfection (data not shown). After successful *in vitro* APP protein modification and Aβ40 reduction, the 20-mer AON was tested in wild-type mice using the same approach as was done for the 33-mer AON. RNA analysis showed dose–response exon skipping in all brain areas of all treated animal groups (Supplementary Fig. S3). Compared with the 33-mer AON RNA data, there was a more efficient exon skip of 70%–80% even in the lowest dose group of 0.5 mg. In cortex, that showed hardly any APP modification in protein level with the 33-mer AON, the 20-mer AON showed a 50%–60% skip. The improved efficiency of the 20-mer AON can be also seen on protein level where there was a clear and more pronounced exon skipping effect compared with protein results with the 33-mer AON. The modified protein reaches 40%–50% of total APP, and can be seen in all brain areas and dose groups (Fig. 5C).

## Discussion

In this study, we show successful and efficient removal of the D-CAA mutation and the y-secretase cleavage site from the APP protein using AON-mediated splice modulation both *in vitro* and *in vivo*.

Neuroblastoma cells, D-CAA patient fibroblasts, and patient neuronally differentiated iPSCs showed efficient exclusion of exon 17 from the APP transcript and generation of the APPΔ17 isoform with a simultaneous lowering of Aβ40 levels. In D-CAA, the mechanism of perivascular Aβ aggregation is still poorly understood. Previous studies have shown that an increase in production of Αβ in D-CAA is not likely the cause of Aβ aggregation [27], which is supported by our results where we found equal levels of Aβ40 and Aβ42 in the cell culture medium of untreated control and patient neuronally differentiated iPSCs. sAPPα is another important APP cleavage fragment that has been found to be neuroprotective, as it stimulates neurite outgrowth, decreases cell adhesion, and increases synaptogenesis [28]. Even though the α-secretase cleavage site is still present in the APPΔ17 isoform, we noticed a decrease in the levels of sAPPα after AON treatment. The sAPPα reduction after AON treatment could be due to limited accessibility of the α-secretase cleavage site to the enzyme. The α-secretase enzyme resides at the plasma membrane surface or in the trans-Golgi network, and part of the transmembrane domain of APP is removed after AON treatment [29]. Future APP cleavage fragment localization studies are needed to investigate the underlying mechanism.

We continued with the *in vivo* proof of concept, by treating wt-mice with the 33-mer and 20-mer AON. Both AONs successfully induced exon skipping on RNA level; however, only the 20-mer AON showed efficient protein modification across all brain regions and doses used. It has been shown that the length of an AON is an important parameter for its activity, and possible side effects seem to be more prominent with increased AON length [24,30]. Moreover, longer AONs are more prone to form secondary structures that interfere with binding to the target sequence and decrease efficiency [31]. Both our *in vitro* data with neuronally differentiated iPSCs and *in vivo* data show that shortening the length of the AON from 33-mer to 20-mer increases the efficiency in protein modification. In future, studies will have to show a treatment effect of APP protein modification in an animal model or other 3D *in vitro* models of the disease, like organoids, to confirm prevention of Aβ40 aggregation and amelioration of the phenotype.

A similar approach of AON-mediated APP modification was studied for Down's syndrome where Chang *et al*. used an 18-mer PS-MOE AON to induce exon 17 skipping of the APP mRNA transcript to lower Aβ production [32] indicating the diversity as well as the reproducibility of this APP protein modification approach, both very crucial aspects in translational research. The novelty of this study is the use of neuronally differentiated patient iPSCs as the most suitable *in vitro* model of the disease, compared with overexpression or fibroblast models that are used by Chang *et al*. This *in vitro* model accurately represents Aβ levels that are similar between healthy people and D-CAA patients. Moreover, this is the first study to show that the APP protein is modified after AON treatment both *in vitro* and *in vivo.*

AONs are a promising asset in the field of therapeutics of central nervous system (CNS) disorders as they can modulate RNA splicing to increase, decrease, or modify protein expression. After an intrathecal injection in humans, AONs can freely distribute throughout the spinal cord and brain, showing efficient cellular uptake, long half-life resulting in limited dosing [33]. Nusinersen is the first CNS AON that has been approved for the treatment of spinal muscular atrophy (SMA) (Spinraza, Biogen) [34,35]. Currently, there are more clinical trials for CNS diseases, such as Huntington's disease, that show promising results [36,37].

Aβ aggregation and deposition around the blood vessels is a characteristic of D-CAA and sporadic CAA. Familial forms of sporadic diseases are crucial in understanding both underlying disease mechanisms and development of therapeutics, because of their early onset, severity of the symptoms, and patient population. In this study, we show that our AON approach is an ideal candidate for the therapy of D-CAA. Success with genetically defined populations could lead in future to similar treatments applied more broadly in diseases where Αβ is a pathogenic driver, such as Alzheimer's disease, familial AD, and sporadic CAA.

## Supplementary Material

Supplemental data

Supplemental data

Supplemental data
