# Supplementary material for: Antisense Oligonucleotide-Induced Amyloid Precursor Protein Splicing Modulation as a Therapeutic Approach for Dutch-Type Cerebral Amyloid Angiopathy
Source: Nucleic Acid Ther. 2021 Oct 12;31(5):351–63. doi: 10.1089/nat.2021.0005 (PMC8823675; doi:10.1089/nat.2021.0005)
Supplement: Supplemental data [file Supp_FigS1.docx]

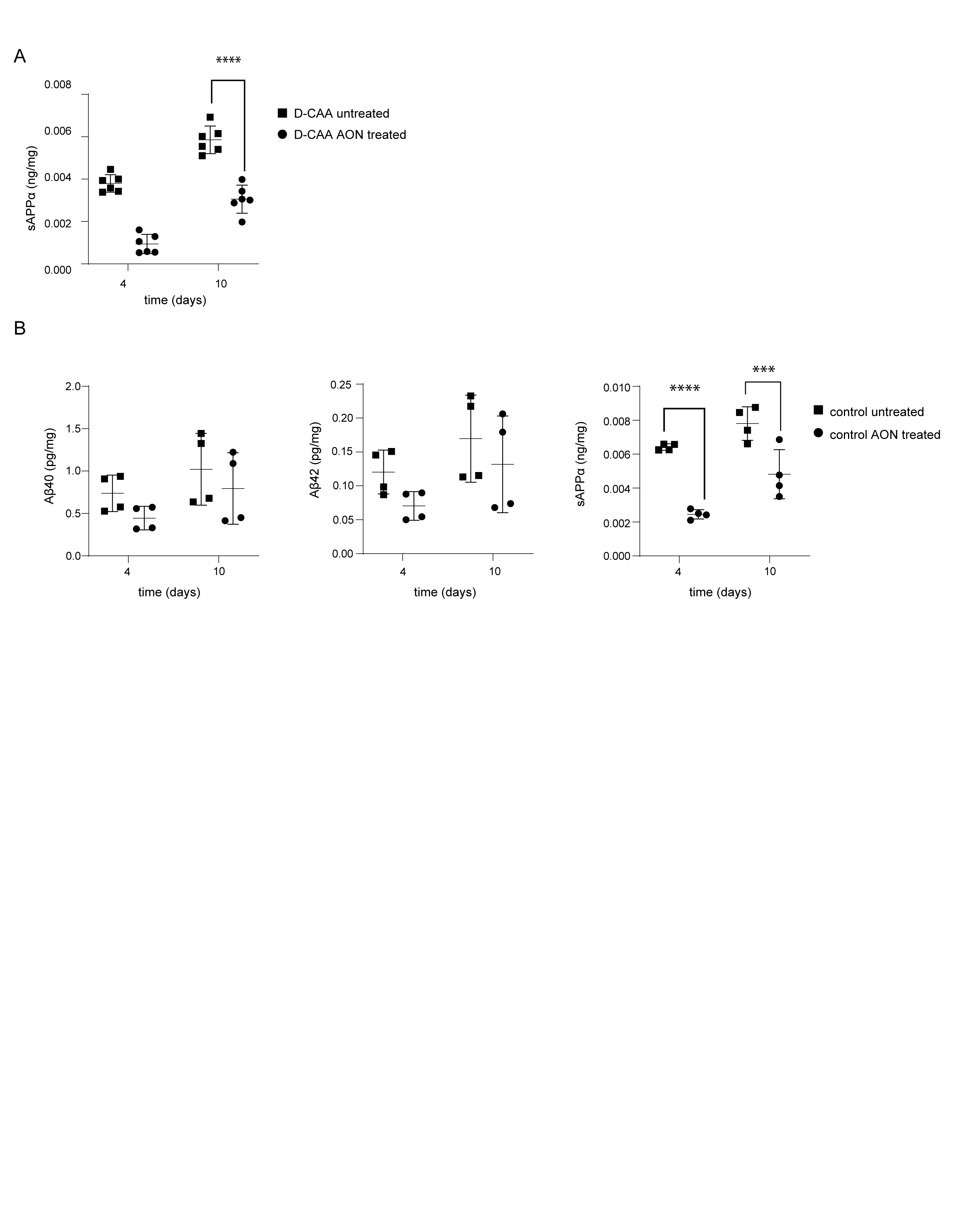
Supplementary data 1. A. sAPPα levels in medium from patient neuronally differentiated iPSCs 4 and 10 days after 33-mer AON transfection, measured with ELISA. sAPPα levels of AON treated cells are significantly lower for both time points compared to untreated cells (±SD; *****P* < 0.0001, two-way ANOVA with Bonferroni’s multiple comparisons test; n = 6). B. Aβ40, Αβ42 and sAPPα levels in control neuronally differentiated iPSCs measured by ELISA. The levels of all 3 cleavage fragments are decreased compared to untreated cells (±SD; ****P*<0.0010, two-way ANOVA with Bonferroni’s multiple comparisons test; n=4).
