# Supplementary material for: Antisense Oligonucleotide-Induced Amyloid Precursor Protein Splicing Modulation as a Therapeutic Approach for Dutch-Type Cerebral Amyloid Angiopathy
Source: Nucleic Acid Ther. 2021 Oct 12;31(5):351–63. doi: 10.1089/nat.2021.0005 (PMC8823675; doi:10.1089/nat.2021.0005)
Supplement: Supplemental data [file Supp_FigS3.docx]

*
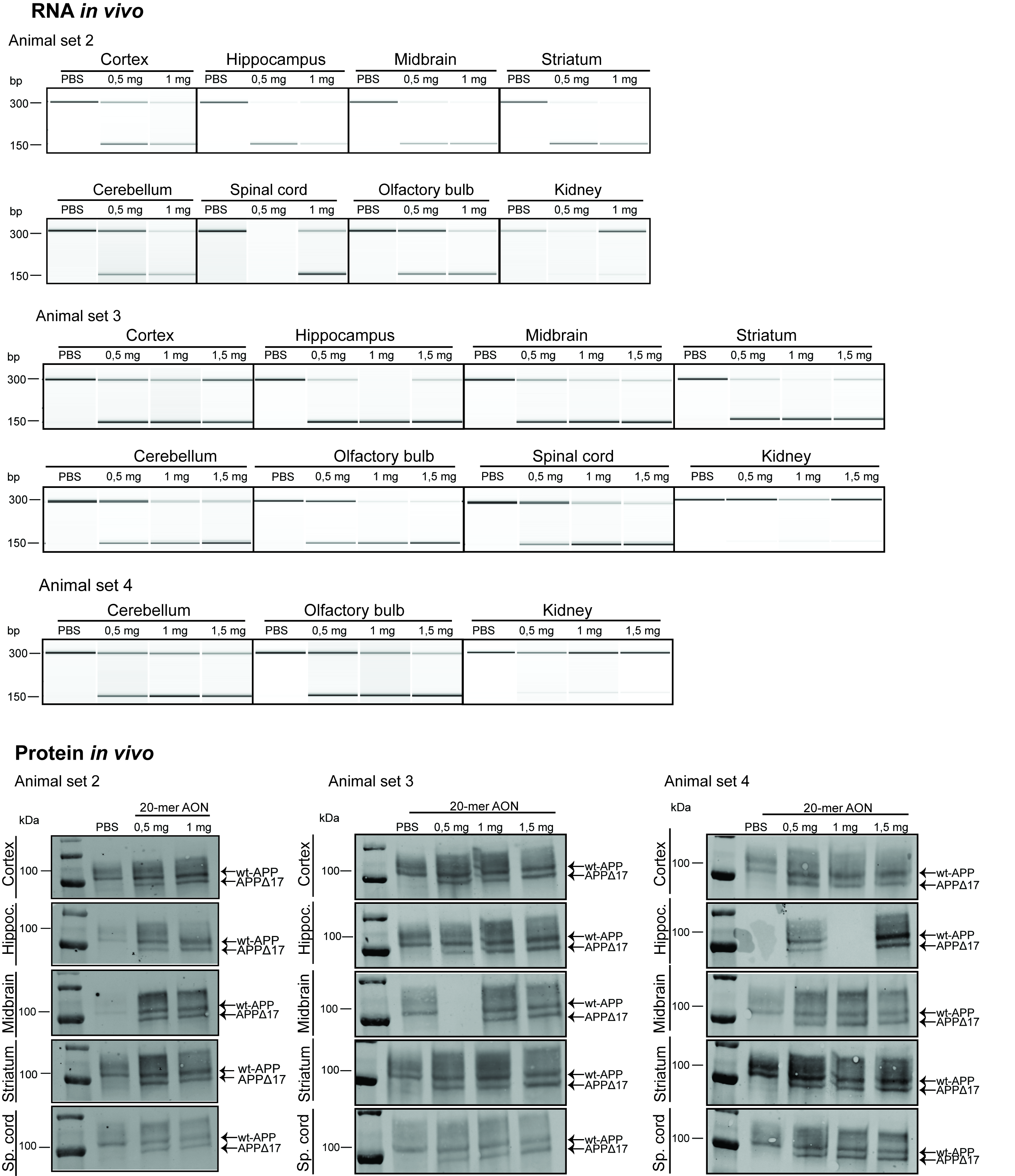
*

Supplementary data 3. *In vivo* RNA analysis of 20-mer AON dose response (0.5 mg, 1 mg and 1.5 mg) treatment in wt-mice.
